# Cellular automata simulation of topological effects on the dynamics of feed-forward motifs

**DOI:** 10.1186/1754-1611-2-2

**Published:** 2008-02-27

**Authors:** Advait A Apte, John W Cain, Danail G Bonchev, Stephen S Fong

**Affiliations:** 1 Department of Chemical and Life Science Engineering, Virginia Commonwealth University, P.O. Box 843028, Richmond, VA 23284, USA; 2 Department of Mathematics and Applied Mathematics, Virginia Commonwealth University, P.O. Box 842014, Richmond, VA 23284, USA; 3 Center for the Study of Biological Complexity, Virginia Commonwealth University, P.O. Box 842030, Richmond, VA 23284, USA

## Abstract

**Background:**

Feed-forward motifs are important functional modules in biological and other complex networks. The functionality of feed-forward motifs and other network motifs is largely dictated by the connectivity of the individual network components. While studies on the dynamics of motifs and networks are usually devoted to the temporal or spatial description of processes, this study focuses on the relationship between the specific architecture and the overall rate of the processes of the feed-forward family of motifs, including double and triple feed-forward loops. The search for the most efficient network architecture could be of particular interest for regulatory or signaling pathways in biology, as well as in computational and communication systems.

**Results:**

Feed-forward motif dynamics were studied using cellular automata and compared with differential equation modeling. The number of cellular automata iterations needed for a 100% conversion of a substrate into a target product was used as an inverse measure of the transformation rate. Several basic topological patterns were identified that order the specific feed-forward constructions according to the rate of dynamics they enable. At the same number of network nodes and constant other parameters, the bi-parallel and tri-parallel motifs provide higher network efficacy than single feed-forward motifs. Additionally, a topological property of isodynamicity was identified for feed-forward motifs where different network architectures resulted in the same overall rate of the target production.

**Conclusion:**

It was shown for classes of structural motifs with feed-forward architecture that network topology affects the overall rate of a process in a quantitatively predictable manner. These fundamental results can be used as a basis for simulating larger networks as combinations of smaller network modules with implications on studying synthetic gene circuits, small regulatory systems, and eventually dynamic whole-cell models.

## Background

Modeling is a means of making predictions and testing our understanding. In some sense, our level of understanding of an entity can be measured by how well we can model that entity [1]. In particular, mathematical modeling has been applied to diverse areas of science [2], including chemistry [3] and biology [4-6]. The quantitative nature of mathematical modeling has the benefit of yielding detailed, objective descriptions and predictions of processes. An accurate mathematical model can help clarify the roles of individual components within a process and generate specific, testable hypotheses and predictions. The quantitative results of a mathematical model also provide an objective basis for evaluating the accuracy of a model when compared to experimental results and can enable iterative improvement of a model [7,8].

One of the main benefits of quantitative modeling and analysis is the ability to identify emergent, general properties. An example of this is that complex networks from the internet to biological metabolism have been found to organizationally function and expand as scale-free networks [9]. Within the scale-free framework, genes, proteins, and metabolites are further organized into functional modules with specific structural motifs [10]. Motifs are defined in terms of graph theory [11,12] as simple connected subgraphs, the abundance of which in a given network is very different from a random graph having the same number of vertices and edges.

One of the most prevalent topological motifs is the feed-forward (FF) loop. Feed-forward loops have been studied in biological systems and have been found to be involved in a number of different processes including regulatory mechanisms [13] and cell differentiation [14]. Transcriptional regulation is found in most organisms and the dynamics of feed-forward motifs in gene regulatory networks has modeled in detail by Alon and co-workers [15-19] to successfully reproduce basic temporal dependencies. Detailed characterization of specific topological motifs should lead to new analyses such as predicting gene regulatory patterns resulting from the aggregation of different topological motifs [20,21]. The mechanism of feed-forward control in the transcriptional network that promotes cell growth was recently elucidated by Palomero et al. [22] and inhibitory feed-forward effects in neural circuits have been studied by Klyachko and Stevens [23]. Cordero and Hogeweg [24] have shown that gene evolution depends on the topology of gene regulatory networks. A recent review [25] analyzed the relation between structural modules and dynamics of cellular networks, as a basis for engineering cells to produce desired properties. More abstractly, studies relating topology to function can lead to better understanding of biological network dynamics such as robust dynamical stability [26] and motif specific spatio-temporal dynamics [27,28]. All of these examples illustrate the connection between biological function and the topology of biological networks.

The present study focuses on the qualitative and quantitative characterization of a variety of feed-forward motifs with different architecture, and the relationship between topology and overall process rate in feed-forward loops. A variety of stochastic simulation methods exist that can be used to model networks dynamics [29-32]. In our study, cellular automata [33] were selected as a promising method for dynamic modeling of all possible topologies of feed-forward loops due to its flexibility, robustness and accuracy. Widely applied in a variety of areas of science and technology, the cellular automata method recently showed great promise for modeling dynamics of complex biological systems [34-38]. In modeling biochemical processes, we followed the general method used by Kier and Cheng [39,40]. Specifically, this study is focused on studying the purely topological effects on motif productivity. Thus, the cellular automata simulations were conducted in a manner so as to keep all probabilistic parameters constant. This effectively "freezes" the process stochasticity, making our simulations *de facto *non-stochastic. Results of cellular automata topodynamic pattern simulations were verified with those obtained by parallel ODE and non-linear differential equations simulations.

## Methods

### Cellular automata modeling of biochemical networks

**Figure 1:**
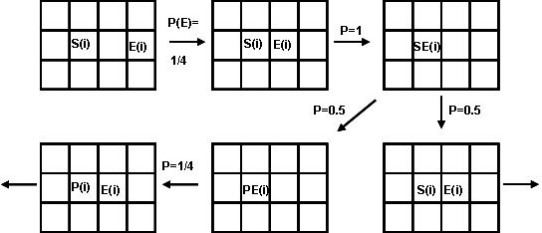
A scheme illustrating the rules that control the dynamics of the enzymatic reaction steps *i*. Here *S*, *P*, *E*, *SE *and *PE *stand for substrate, product, enzyme, substrate/enzyme and product/enzyme, respectively. The probability of motion is 1/k, *k *= 1 to 4 being the number of unoccupied neighboring cells; probability equal to 1 is postulated for *S*(*i*) and *E*(*i*) to join, as well as for *PE*(*i*) to disjoin. The transitional probability for the *PE*(*i*) formation is assumed equal to 0.5, which makes the first reaction step reversible.

Cellular automata (CA) are modeling tools that represent dynamic systems discretely in space, time, and state. The overall system behavior is specified entirely by rules governing local relationships. In the most common 2D-version, CA models are constructed on a grid of squares called cells. The grid size may vary considerably, depending on the system. To eliminate any boundary effects, the grid is usually built on the surface of a torus. In our study we used lattices within the range of 100 × 100 to 220 × 220 (*fide infra*). The following rules were employed (See Fig. 1 for an illustration of the most essential probabilistic rules):

• Space and time are discrete: There is a two-dimensional toroidal grid of cells that is viewed at subsequent equidistant time steps.

• At each time step, each cell has a single state – empty or occupied. The cell may be occupied by an enzyme, a substrate, a product, a substrate/enzyme complex or a product/enzyme complex.

• The state of a cell at a given time step depends only on its own state and the cell states in its neighborhood, all taken at the previous step.

• Each cell has four sites (von Neumann neighborhood), on which interactions can be simulated.

• The contents of a cell may break away from an occupied cell or move to join a cell that is occupied. The probabilities for moving, P_M_, joining, P_J _(XX) and P_J _(XY), and breaking away, P_B_(XX) and P_B_(XY), are P_M _= P_J _= P_B _= 1. This means that all cells may move, join, and break apart with equal probability.

• The overall probability of a movement, P_M _= 1, is divided into probabilities for movements onto *k *grid directions, where *k *= 1–4 is the number of unoccupied neighboring cells.

• The only joining of two cells that has a consequence is that between the specific substrate S(i) and the specific enzyme E(i). When such an encounter occurs, a substrate-enzyme complex (SE(i)) is formed. This complex has an assigned transitional probability, P_T _= 0.5, of changing to a new product-enzyme complex PE(i).

$$\text{S}(\text{i})+\text{E}(\text{i})↔\text{SE}(\text{i})\overset{{\text{P}}_{\text{T}}}{\to }\text{PE}(\text{i})\to \text{P}(\text{i})+\text{E}(\text{i})$$

In such reactions, the transitional probability is regarded as a measure of enzyme activity or propensity, and may be varied within the entire range of values between 0 and 1. In this study, P_T _was uniformly assumed to be 0.5 to allow reversibility of the first reaction step and to isolate the purely topological effects on motif productivity. By assuming the movement, joining and breaking probabilities are all equal to 1, and by fixing the only essential probabilistic parameter, transitional probability, to a constant value (0.5), we "freeze" the model stochasticity, and have *de facto *a non-stochastic CA modeling.

The rules, applied at random to all cells, represent one iteration of the modeling procedure, which determines the cells new states and trajectories. A cell and its four-cell environment can acquire any of the five states defined above. The initial state of the system is random and, thus, does not determine subsequent configurations at any iteration. After many iterations, the system reaches a relatively constant configuration (analogous to a chemical steady state), characterized by counts of cells. The model is statistical; many runs are performed, and the number of iterations needed to attain a steady-state is averaged. The specific CA parameters: number of runs, number of cells for different species, grid density, and percent of conversion of the source substrate into the target product were optimized, as discussed in RESULTS.

### Differential equations modeling of networks

**Figure 2:**
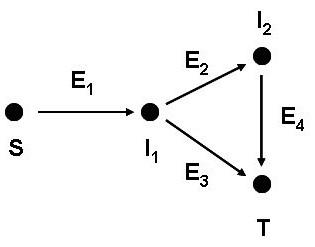
Example of a feed-forward motif with four vertices and four edges, explaining the notation used in the linear differential equations. *S*, *T *and *I *stand for source, target and intermediate node, respectively.

Ordinary differential equations (ODEs) were used to model select topological motifs to compare with results from CA simulations. The simplest way to construct such an ODE model is to treat each feed-forward link A→B without regard to the underlying biochemical processes (e.g., formation of substrate-enzyme complexes and subsequent dissociation of the complexes). In doing so, we neglect any nonlinear interactions of various species. Each link has an associated rate constant and each vertex in the feed-forward motif gives rise to an ODE. For the system shown in Fig. 2, the ODEs are:

$$\frac{dS}{dt}=-k_{S1}S$$

$$\frac{dI_{1}}{dt}=k_{S1}S-(k_{12}+k_{1T})I_{1}$$

$$\frac{dI_{2}}{dt}=k_{12}I_{1}-k_{2T}I_{2}$$

$$\frac{dT}{dt}=k_{1T}I_{1}+k_{2T}I_{2}$$

Importantly, the resulting constant-coefficient linear systems of ODEs can be solved explicitly, yielding exact formulas for each state variable. In particular, the formula describing the evolution of the target state (T) can be used to determine how much time is needed to achieve a specified level of conversion. One may then study the effect of network topology on the dynamics by ranking the conversion times for various feed-forward motifs, starting from the same initial conditions.

We also attempted to more carefully model the biochemical processes underlying each feed-forward link in Fig. 2. This time the biochemical processes underlying each feed-forward link is presented in detail, tracking the amount of each species, the amount of each enzyme, and the amount of each substrate-enzyme complex. Thus, assuming irreversibility of all reaction steps, we get one ODE for each vertex and two ODEs for each edge in the motif. Assuming mass-action kinetics in the formation of substrate-enzyme complexes introduces nonlinearity. The motif in Fig. 2 is thus described by the set of equations:

$$S+E_{1}\overset{k_{1}}{\to }SE_{1}\overset{{\bar{k}}_{1}}{\to }E_{1}+I_{1}$$

$$I_{1}+E_{2}\overset{k_{2}}{\to }I_{1}E_{2}\overset{{\bar{k}}_{2}}{\to }E_{2}+I_{2}$$

$$I_{1}+E_{3}\overset{k_{3}}{\to }I_{1}E_{3}\overset{{\bar{k}}_{3}}{\to }E_{3}+T$$

$$I_{2}+E_{4}\overset{k_{4}}{\to }I_{2}E_{4}\overset{{\bar{k}}_{4}}{\to }E_{4}+T$$

and yields twelve ODEs, matching the detailed mass-action kinetics. This system can be effectively reduced to a system of eight ODEs, since the rate of formation of a substrate-enzyme complex is the negative of the rate of change of the enzyme concentration. An example is given in eq 10:

$$\frac{d\left[I_{1}\right]}{dt}=-k_{2}\left[I_{1}\right]\left[E_{2}\right]-k_{3}\left[I_{1}\right]\left[E_{3}\right]+{\bar{k}}_{1}\left[SE_{1}\right]$$

where *k*_1 _through *k*_4 _and ${\bar{k}}_{1}$ through ${\bar{k}}_{4}$ denote rate constants (which for extracting the topological effects on motif productivity are assumed equal) and [*E*_1_] through [*E*_4_] denote enzyme concentrations. Because nonlinearity prevents solving the ODEs explicitly, they were solved numerically by the forward Euler method with a time step of 0.001 units. There was no need to use a more sophisticated numerical method as we considered the special case in which all rate constants are equal, and the system is not stiff (the eigenvalues of the Jacobian matrices associated with these systems of ODEs are (i) all negative and (ii) are of the same order of magnitude).

We have also studied the possibility for the first "half" of each process to be reversible. Here, one must track the amount of (i) the four species; (ii) the four enzymes; and (iii) the four substrate-enzyme complexes. This adds more terms to the nonlinear system of eight ODEs, as illustrated by the last two terms in eq.11

$$\frac{d\left[I_{1}\right]}{dt}=-k_{2}\left[I_{1}\right]\left[E_{2}\right]-k_{3}\left[I_{1}\right]\left[E_{3}\right]+{\bar{k}}_{1}\left[SE_{1}\right]+r_{2}\left[I_{1}E_{2}\right]+r_{3}\left[I_{1}E_{3}\right]$$

where *r*_*i *_stands for the rate constant of the reversed reaction step *i*.

## Results

### Selection of the major CA parameters

**Figure 3:**
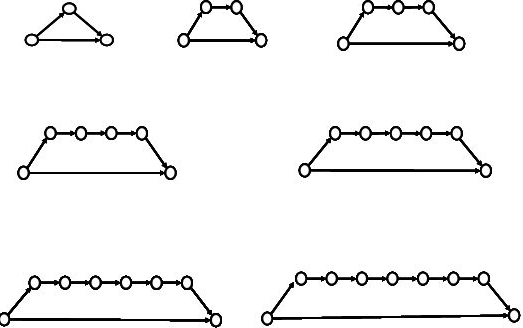
The members of the examined primary feed-forward motif series FFA.

To generate accurate and reproducible results, several series of tests were performed to optimize the basic CA parameters to be used before proceeding with the detailed study on feed-forward motifs. Due to the statistical nature of the CA modeling, a large number of simulation runs should be performed to obtain statistically-meaningful results. The optimal number of runs must be large enough to provide reliable statistics, and at the same time not excessively large so as to minimize the computation time. We examined the influence of the number of runs on the number of iterations needed to reach 100% conversion of the source substrate into the target product (Table 1). The tests were performed at different degrees of lattice occupancy (termed *lattice density*) and with different number of nodes (3, 6 and 9) in the feed-forward FFA series shown in Figure 3. Results for 50 runs differed from those obtained at 100, 250 and 1000 runs (Table 1); the latter three were practically the same and within the range of standard deviation observed. Therefore, 100 runs were selected to perform the basic feed-forward modeling.

**Table 1:** Cellular Automata simulation of the dynamics* of feed-forward series FFA shown in Fig. 1

Number of Nodes	Lattice Density %	Number of Runs				SD** average	SD %
		50	100	250	1000		

3	3.6	447	469	458	460	7	1.51
	10	143	145	144	144	8	5.56
	60	21	20	21	20	8	39.0
6	3.6	939	895	893	906	8	0.89
	10	287	293	290	288	10	3.45
	60	41	42	41	41	9	21.6
9	3.6	1430	1425	1426	1422	2	0.14
	10	458	460	456	458	2	0.44
	60	65	65	65	65	9	13.9

* Dynamics is expressed as the average number of iterations needed for 100% conversion of 100 source substrate cells into the target product. It is modeled at different number of nodes, different lattice densities, and different number of cellular automata runs.

** Standard deviation

**Figure 4:**
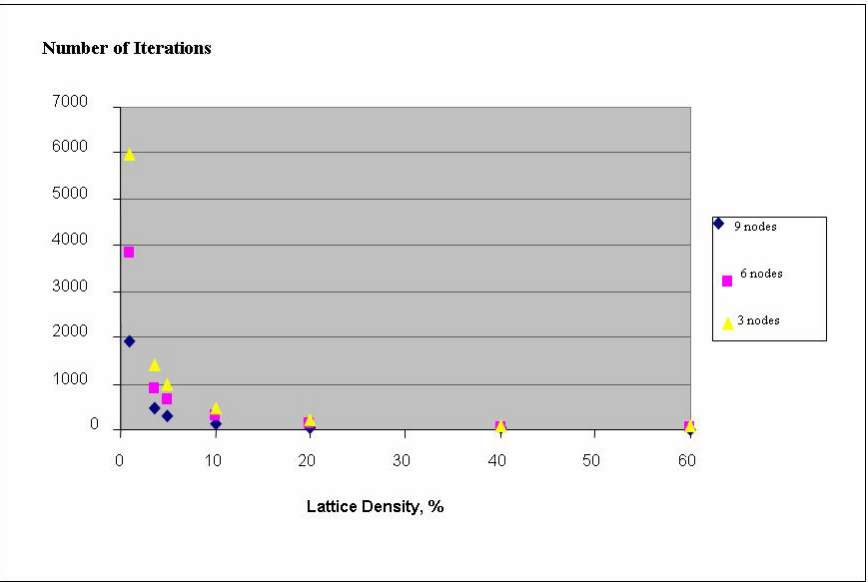
The overall rate of the feed-forward process of the source substrate conversion into the target product in the Feed-Forward series A (FFA) decreases rapidly with lattice density. The number of iterations, needed to attain a steady-state in Series 1, 2 and 3, correspond to the number of feed-forward nodes (Fig. 3) equal to 3, 6, and 9, respectively. All data are averaged over 100 runs simulation.

Another parameter investigated was the CA lattice density (the number of cells per unit area). A high density can impede the free cell motion and the results obtained in different runs could diverge considerably. That was confirmed in our tests, which showed over 25-fold increase in the standard deviation of the number of iterations when the lattice density was increased within the series 1.0, 3.6, 5, 10, 20, 40, and 60%. On the other extreme, a very low density (See Figure 4) would unnecessarily prolong the time for attaining a steady state. For these reasons we selected the 3.6% lattice density (corresponding to 100 × 100 cells lattice with a total of 360 cells occupied by substrates and enzymes) as a constant parameter for the detailed study of the dynamics of feed-forward motifs. This required resizing the lattice for each of the FF motifs examined. All lattice sizes used were within the range of 100 × 100 to 220 × 220 cells.

More detailed simulations were run to quantitatively capture the manner in which the feed-forward dynamics vary with different lattice density, *D*, and the number of nodes, *V*, in the FFA-series. Equation (12), relating these quantities, was derived from the data of Table 1:

*I *= 640.5*VD*^-1.0862 ^- 8.281ln(*D*) + 36.08

The equation derived shows considerable acceleration of the FF processes with the increase in density in agreement with the law of mass action, since density is proportional to a substrate concentration modeled as number of cells per unit lattice area.

In deriving Eq (12) we used the linear dependence of the number of iterations on the number of FF loop nodes: I = aV + b, which for V = 3, 6, and 9 nodes were obtained with correlation coefficient R^2 ^= 0.9961, 0.9988, and 0.9998, respectively. The regressions best expressing the dependence of a and b on the lattice density *D *were

*a *= 640.5*D*^-1.0862 ^and *b *= -8.281ln(*D*) + 36.08

with R^2 ^equal to 1.0000 and 0.9901, respectively.

The last parameter to select was the number of source cells (*N*). The number of cells of all other pathway constituents was kept equal to 100 cells. The enzymes associated with each of the biochemical reaction steps were kept equal to 20 cells. All enzyme activities were also kept equal (by applying the same CA probabilistic rule) in order to extract the purely topological effects on the network dynamics Since the concentration, simulated as number of cells per unit lattice, was kept constant, *N *itself should not influence the correlation coefficient of the linear model relating the number of nodes in the pathway to the rate of the source-to-outcome conversion. As shown in Table 2, while the correlation coefficient remains within the same range, the increase in the number of source cells in the feed-forward motif reduces the model standard deviation at the cost of considerable increase in the number of iterations needed to reach a steady-state. As a reasonable compromise between a low standard deviation and too many iterations, we chose to perform the detailed study in the next section at N = 500, a parameter value that enabled us to attain steady-state with an average standard deviation of 0.31% for less than 10,000 iterations.

**Table 2:** Influence of the size of the feed-forward motif on the dynamics of 100% conversion of the source substrate into target product

N*	Iterations Range	R^2^	SD %
100	469 – 1425	0.9960	0.99
300	1603 – 4473	0.9994	0.76
500	2966 – 8444	0.9996	0.31
700	4435 – 13282	0.9989	0.28
900	6397 – 20526	0.9931	0.19

*N – Number of source nodes; R^2 ^– correlation

coefficient; SD % – relative standard deviation

### Modeling seven feed-forward series having different topology

**Figure 5:**
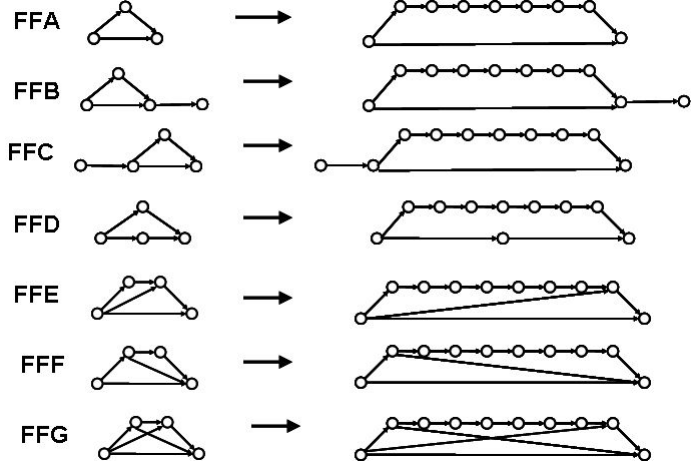
Feed-forward motifs with different network topologies. The first and the last member of the examined seven feed-forward motifs series are shown only. The series include all structures with intermediate number of nodes (FFA series had networks with 3 to 9 nodes; all other series had 4–10 nodes).

The feed-forward motif FFA shown in Fig. 3 may be termed the *primary feed-forward *series (PFF) to be distinguished from some more complex variations on the same feed-forward pattern. One may also consider double, triple, etc., FF motifs, which include secondary, tertiary, etc. feed-forward edges. Such structural motifs with more complex topologies may be obtained by merging two or more primary FF motifs. This approach may be of interest in predicting dynamic patterns in larger networks as combinations of well established patterns in small subnetworks (motifs). We selected for our study several such complex cases of feed-forward patterns of PFFs (Fig. 5).

Two versions of CA model results were compiled, 50% conversion and 100% conversion. These two categories of results represent the number of CA iterations needed for 50% and 100% conversion of the source substrate into the target product. This number serves as an inverse measure of the FF motif dynamics (the larger the number of iterations needed for arriving at a steady-state, the slower the overall process). As seen from Table 3, the linear regression models obtained for 50% conversion have lower correlation coefficients (R^2 ^= 0.9796 to 0.9980) and considerably higher standard deviations (SD = 1.70 to 4.14%), as compared to the corresponding models with 100% conversion (R^2 ^= 0.9990 to 0.9996 and SD = 0.25 to 0.50%). Based upon these results, the subsequent analyses are based on the results obtained with 100% conversion only.

**Table 3:** Linear dependence of the overall rate of feed-forward motifs on the number of motive nodes

Series	N	Iterations range	Regression	R^2^	SD %
FFA-50	3–9	804 – 2160	219.00 N + 227.14	0.9796	4.14
FFB-50	4–10	1735 – 3736	320.18 N + 483.46	0.9966	1.43
FFC-50	4–10	2043 – 4530	402.36 N + 481.36	0.9980	1.71
FFD-50	4–10	1375 – 3428	329.93 N + 148.50	0.9939	2.38
FFE-50	4–10	1080 – 2247	202.93 N + 258.64	0.9874	2.62
FFF-50	4–10	785 – 1574	133.36 N + 235.07	0.9981	1.70
FFG-50	4–10	1955 – 4104	365.61 N + 395.89	0.9900	2.78

FFA-100	3–9	2966 – 8444	920.75 N + 187.50	0.9996	0.31
FFB-100	4–10	4187 – 9044	800.93 N + 998.07	0.9994	0.29
FFC-100	4–10	4542 – 10565	993.71 N + 556.00	0.9990	0.25
FFD-100	4–10	3291 – 8067	802.64 N + 89.357	0.9993	0.34
FFE-100	4–10	3505 – 7599	683.57 N + 766.14	0.9994	0.33
FFF-100	4–10	2408 – 4927	418.21 N + 705.07	0.9991	0.50
FFG-100	4–10	2721 – 5086	397.61 N + 1121.8	0.9991	0.42

The overall rate is measured by the number of iterations needed for 50% and 100% conversion of the source substrate into the target product. Seven series (Fig. 3) of feed-forward motifs with different topology, constant number of source cells, *N *= 500, and constant lattice density, *D *= 3.6%, are studied.

## Discussion

**Figure 6:**
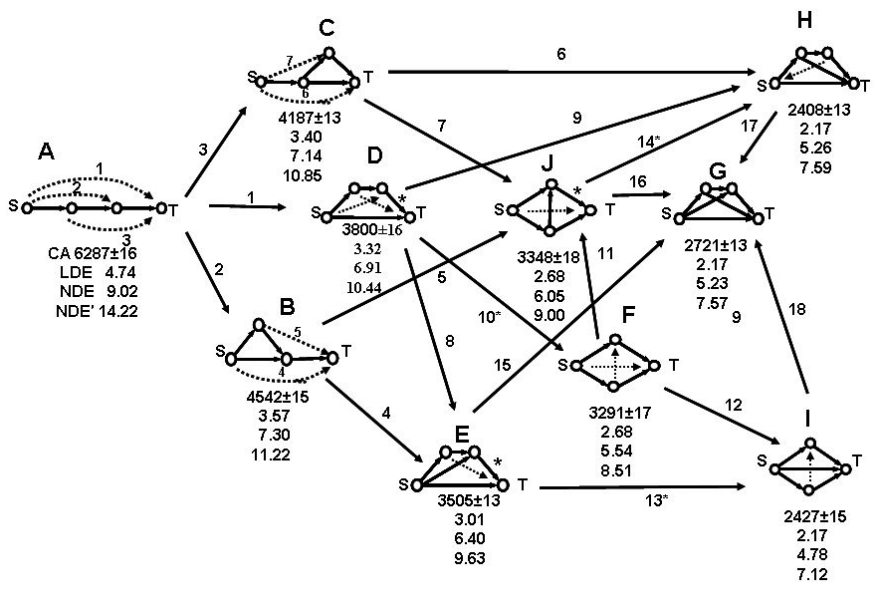
Chart of all ten four-node motifs (eight feed-forward and **F **– biparallel) of conversion of the source node S to target node T: **A **– linear, **B**, **C**, **D**, **E**, **G **and **H **– feed-forward, **F **and **J **– bi-parallel, and *I *– tri-parallel. The motifs are ordered according to their dynamic efficacy in producing the target product with a highest rate, assessed by decreasing number of iterations, and decreasing time, as produced by the linear (LDE) and nonlinear ODE models for 90% S → T conversion. The nonlinear irreversible and reversible ODE times are denoted by NDE and NDE', respectively. The mutual transformations of the structures shown include 15 additions of another feed-forward link, whereas those marked by an asterisk (**D → F**, **E → I**, and **J → H **conversions) include a reversal of a single link direction.

The central focus of our analyses was to study how network topology affects the dynamics of processes in different feed-forward motifs. In order to ensure that the networks analyzed were comparable to enable the identification of stable structure-dynamics patterns, we assumed that (i) the rate constants for all processes are equal, (ii) the initial conditions are chosen such that the source (S) is initially five times larger than each of the other species, and (iii) all enzyme activities are constant and equal. We constructed a chart containing all ten motifs having four nodes (Fig. 6) and their mutual transformations (15 additions of an edge with the formation of a new cycle, and three link direction reversals connecting feed-forward motifs with bi- and tri-parallel ones), and performed both CA and ODE linear and nonlinear modeling. Each network gives rise to a system of four linear ODEs, which can be solved explicitly. In the nonlinear case we performed numerical simulation with both irreversible and reversible first reaction steps. In all versions of the ODE models the networks were ranked according to their 90% conversion times. The numerical ODE values stand for the time measured in arbitrary units needed for a 90% overall conversion of the source substrate *S *into the target product *T*.

The comparison of the efficacy of performance of the ten four-node networks (Fig. 6) shows that CA and linear ODE order the motifs in the same way with a minor exception. Namely, CA ranks structures **H **and **I **with the same highest conversion rate (2408 ± 13 and 2427 ± 15, respectively), since the iteration numbers overlap within the range of their standard deviations. The linear ODE also ranks **H **and **I **as the fastest four-node topologies, adding a third structure **G**, not only showing the same conversion time 2.17, but this time T(t) is given by the exact same formula in all three cases (eq. 17).

The nonlinear ODE models with reversible and irreversible first steps produce identical ordering of the ten structures. It coincides with the ordering of the first seven structures, described above by CA and linear ODEs, while suggesting that network **I **is the fastest, **G **and **H **having very close performance, **H **shown as slightly slower than **G**.

The topological analysis of the nine networks revealed some useful patterns of their dynamics. Although the networks analyzed here are relatively simple, they could be of use when analyzing local topology in large complex networks. Several of the observed topodynamic patterns are described below.

### Dynamic Feed-Forward Pattern 1 (DFFP1)

The shorter the graph distance d(S→T) between the source node and the target node in a feed-forward motif, the higher the overall conversion rate:

**A(d = 3) < B, C (d = 2) < D, E, G, H, I (d = 1)**

Note, that the bi-parallel motifs **F **and **J **do not obey this rate inequality.

### Dynamic Feed-Forward Pattern 2 (DFFP2)

The shorter the average path length *l*(S→T) between the source node and the target node in a feed-forward motif, the higher the overall conversion rate:

**A (*l *= 3) < B, C (*l *= 2.5) < D, E, G, H (*l *= 2) < I (*l *= 5/3)**

Accounting for all S → T paths is a slightly more sensitive pattern, which singles-out network **I **as the most efficient four-node structure, in agreement with the result obtained by the nonlinear ODE model. The bi-parallel motifs **F **and **J **do not obey this feed-forward topodynamic trend, which is more important in larger networks where the number of S → T paths increases rapidly.

### Dynamic Feed-Forward Pattern 3 (*Isodynamicity*)

Some feed-forward motifs with different topology produce the same overall S → T conversion rate by the CA and linear ODE models:

**CA: H (2408 ± 13) = I(2427 ± 15)**

**ODE: G = H = I = 2.169053700**

Eq. (16b) follows from the analytical solution of the linear differential equations for structures **G**, **H**, and **I**:

$$T(t)=1-\frac{7}{8}e^{-t}$$

The linear ODEs also classify motifs **F **and **J **as isodynamic, obeying the same kinetic equation:

$$T(t)=1-\frac{3}{2}e^{-t}+\frac{5}{8}e^{-2t}$$

The CA and the nonlinear ODE simulations showed these two motifs with different, although relatively close efficacy, the structure **J **being the slower one:

**CA: J (3348 ± 18) < F (3291 ± 17)**

**Irreversible Nonlinear ODE: J (6.05) < F (5.54)**

**Reversible Nonlinear ODE: J (9.00) < F (8.51)**

**Linear ODE: J(2.679) = F(2.679)**

The property of isodynamicity is a surprising novel network pattern, which could warrant further detailed studies.

### Dynamic Feed-Forward Pattern 4 (DFFP4)

**Figure 7:**
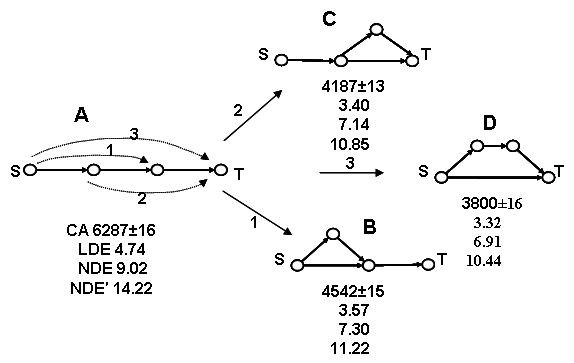
Topological feed-forward transformations (1, 2, and 3) always accelerate processes described as a linear chain of events. Different mechanisms of ring closure are shown, the fastest topology being the one with a direct Source → Target feed-forward link. The linear and nonlinear irreversible and reversible ODE times are denoted by LDE, NDE and NDE', respectively.

**Figure 8:**
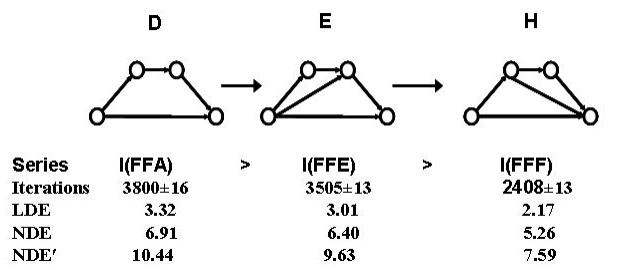
Adding a second feed-forward edge always accelerates the processes in a feed-forward motif. There is no such general pattern for the addition of a third feed-forward link (Compare **E → G **to **H → G**). The linear and nonlinear irreversible and reversible ODE times are denoted by LDE, NDE and NDE', respectively.

Any ring closure of a linear chain of conversion of a source substrate *S *to the target product *T *accelerates the transformation. Acceleration of the process is strongest when the feed-forward link directly connects the substrate to the target and is the smallest when the link connects the substrate with an intermediate product (Figs. 7, 8).

**A < B < C < D**

The ring-closures described by this pattern are shown in Fig. 7 with serial numbers 1, 2, and 3. The generality of this topology-dynamics relationship was verified for the entire series FFA, FFB, and FFC (Fig. 5) having up to ten motif nodes. In all cases, the standard deviation the number of CA iterations was found to be more than two orders of magnitude smaller than that number. This pattern goes beyond the simple topological patterns 1 and 2 shown above, which cannot discriminate between FFB and FFC series.

### Dynamic Feed-Forward Pattern 5 (DFFP5)

Adding a second feed-forward edge (*double feed-forward motif*), between any pair of nodes in the longer path of the FF loop, speeds up the dynamics of the source substrate conversion into the target product (Fig. 8).

**D < E < H**

These inequalities for the number of iterations, illustrated in Fig. 8 with four node motifs, were verified and found valid with no exceptions for all sizes of the three FF series examined (four to ten loop nodes). Comparing the FFF and FFE series, one may generalize that the acceleration of substrate-to-target conversion is higher when the second FF-link starts in a node located on the longer source-target path and ends into the target node, rather than to start in the source node and end in another node before the target one. Since the structures of the triple-feed-forward motif FFG (see graph **G **in Fig. 8) combine the CA trends of the FFF and FFE series, the acceleration in this series is intermediate between the ones of FFE and FFF. However, the ODE models do not confirm this result with the linear model showing G and H to be isodynamic, whereas the two nonlinear models shows G as slightly more efficient than H. Therefore, adding a third feed-forward link does not necessarily result in acceleration and no stable trend exists.

### Dynamic Feed-Forward Pattern 6 (DFFP6)

**Figure 9:**
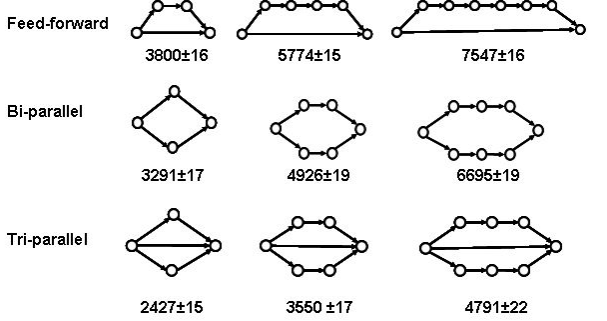
At the same number of nodes, the feed-forward motif is slower than the bi-parallel motif. The topology producing the fastest dynamics is that of the tri-parallel motif **I**.

Reversing the direction of one or more links in a feed-forward motif to turn it into a bi-parallel and tri-parallel one increases the network efficacy. (Figs. 6, 9).

**Feed-Forward < Bi-Parallel < Tri-Parallel**

Three such conversions:

**D < F**, **E < I**, **J < H**

are shown in Fig. 6, where they are denoted by asterisks.

## Conclusion

The technique of dynamic modeling with cellular automata shows great promise in modeling complex biological systems. Such systems can be broken down to subsystems of smaller scale (to ease computational time) and simulated independently so as to shed light on the processes on a larger scale. The essential element in such applications is the extraction of useful topological-dynamic (topodynamic) patterns, which identify specific effects of topological structure on the dynamics of network processes while keeping all kinetic parameters constant. The beauty of the topological approach in studies of dynamics is in the generality of the patterns found, which are independent on the nature of the processes, and may be applied to any process of chemical transformation, as well as to any process of mass, energy or information transfer down the forward direction of the motifs.

The dynamics of the feed forward motifs observed in this study revealed important aspects of networks with such components. Not only does any feed-forward link added to a linear cascade of chemical/biochemical reactions accelerate the process, but the acceleration is further enhanced by adding a second forward link in the feed-forward loop. The topological hierarchy established in this study for four-node motifs predicts that the acceleration of the overall process in such motifs continue increasing with the decrease in the distance (both along the shortest path and along all paths) between the input and output nodes, whereas at the same distance the cellular automata and differential equation simulations produce in a similar manner a further distinction between the motifs dynamic efficacy. The intriguing property of isodynamicity was identified showing motifs with the same number of nodes and different topology to have the same overall rate of input-to-output transformation. If shown to be present in larger biological networks, the observed isodynamic property could indicate a level of biological robustness at a topological level. Further topology-dynamics studies involving construction of networks from combinations of such structural blocks will aid in increasing our understanding of complex biological networks.

## Abbreviations

CA – Cellular Automata, ODE – Ordinary Differential Equations, FFP – Feed-Forward Pattern, FFA through FFG – Feed-Forward Series A through G

## Competing interests

The author(s) declare that they have no competing interests.

## Authors' contributions

A. Apte performed the cellular automata simulations, J. W. Cain did the ODE modeling, D. Bonchev and S. Fong planned the study, and provided the topological (D.B.) and the background (S.F.) analysis. All authors have read and approved the final manuscript.
